# Customizing Multicolored Orbital Angular Momentum Combs

**DOI:** 10.1021/acs.nanolett.5c00467

**Published:** 2025-03-24

**Authors:** Hammad Ahmed, Muhammad Afnan Ansari, Rong Yan, Xianzhong Chen

**Affiliations:** † Institute of Photonics and Quantum Sciences, School of Engineering and Physical Sciences, Heriot-Watt University, Edinburgh EH14 4AS, U.K.; ‡ MIIT Key Laboratory of Complex-field Intelligent Sensing, Beijing Institute of Technology, Beijing 100081, China

**Keywords:** optical metasurfaces, orbital angular momentum combs, orbital angular momentum spectra, complex structured beams

## Abstract

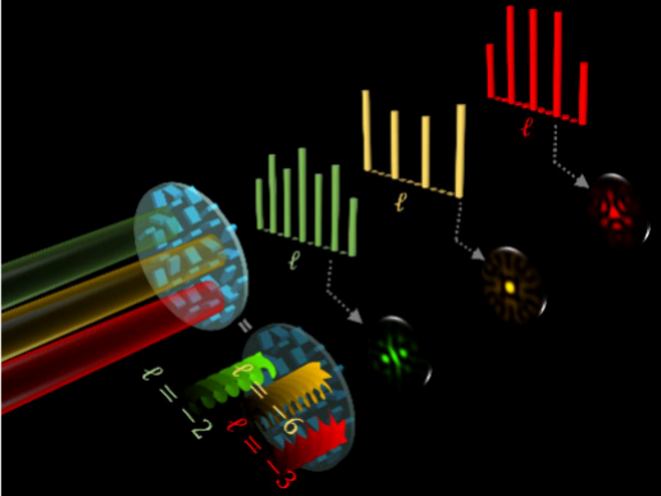

Current orbital angular momentum (OAM) combs generating technology is hindered by bulky optical systems, limited control, and lack of multicolored information, impeding system integration and practical applications. We present a metasurface approach to realizing multicolored OAM comb engineering along the light propagation direction. The OAM combs are measured based on the intensity of bright spots in the generated intensity patterns that correspond to the weights of the OAM modes. Three OAM combs with different colors are generated at different observation planes. The positioning of transition points along the azimuthal direction is the key to shaping the OAM distribution of the generated beams. OAM combs with customized mode spacings and broad OAM spectra are obtained. Our approach provides a compact platform to realize OAM combs with multidimensional information in the domains of the OAM spectra, frequency, and space, which can significantly enhance the information capacity for potential applications in optical communications.

An optical vortex (OV) beam is characterized by its helical phase profile described by exp(ilφ), where *l* and φ are the topological charge (TC) and the azimuthal angle, respectively. This phase profile leads to the formation of a doughnut-shaped ring with a transverse component of Poynting vectors. The azimuthal phase dependence of an OV beam carries an orbital angular momentum (OAM), which has been widely used in various fields such as optical communications,^1,2^ optical imaging,^3^ particle manipulation,^4^ and OAM microlasers.^5^ An OAM comb of light refers to a complex structured light beam that simultaneously carries multiple discrete and equally spaced OAM modes.^6^ Recently, the use of OAM combs has gained considerable interest due to their unique optical properties and potential applications. For example, OAM combs were applied in OAM multicasting, facilitating multiplexed data transmission and significantly enhancing the information capacity of OAM-based optical communications.^7,8^ An OAM comb was also used as a flexible key^9,10^ in holographic encryption and decryption. To improve security and efficiency in quantum communications, we can use OAM combs to create multidimensional photon entanglement. However, current methods for generating and tailoring OAM combs rely on adaptive modification,^8^ pattern-search algorithm,^11^ mode iteration,^12^ and bulky optical elements such as nested ring cavity,^6^ spatial light modulators,^13^ and pinhole plates.^14^ These approaches are not only complex and costly but also impose a significant computational burden due to long calculation times. Therefore, there is a pressing need for a noniterative, compact, and flexible approach to engineering OAM combs.

Optical metasurfaces, precisely designed and patterned planar nanostructures, can manipulate the amplitude, phase, and polarization of light at the subwavelength scale,^15,16^ revolutionizing conventional optical design to develop ultrathin optical elements with unusual functionalities. Optical metasurfaces have been used to realize various structured light beams with unique optical features,^17−19^ including Ince-Gaussian beams,^20^ grafted vortex beams,^21,22^ vector vortex beams (VVBs),^23,24^ OAM holograms,^25,26^ spatiotemporal beams,^27,28^ Airy beams,^29−31^ polarization knots,^32,33^ and edge imagers.^34−36^ Recently, there is an attempt to generate OAM combs at a single wavelength in the THz domain,^37^ which lacks multicolored information and restricts the number of accessible OAM modes. Furthermore, the THz implementation is less suitable for compact integration into optical communication systems and quantum applications due to the relatively large device size and the constraints imposed by the THz wavelength scale. The generation and manipulation of multicolored OAM combs in the visible range in a sequential arrangement remain extremely challenging due to both technical and fundamental limitations. Multicolored manipulation and sequential control introduce additional degrees of freedom, offering enhanced control over mode spacing, spectral breadth, and spatial distribution—capabilities that are highly desirable for various applications.

To address the challenges in the generation of the OAM combs, we propose and experimentally demonstrate a metasurface platform to generate and manipulate the OAM combs. Three OAM combs with different colors are generated at different observation planes. The concept of azimuthal binary gratings (phase only) is utilized to design on-demand OAM combs.^13^ Multiple transition points are integrated in a spiral phase along an azimuthal direction to engineer an OAM comb. The position and number of transition points are carefully controlled to generate an arbitrary OAM comb. To the best of our knowledge, this is the first demonstration of the use of a single metasurface to generate multicolored OAM combs with complicated OAM spectra at various predefined observation planes. Multiple colors in these OAM combs along the direction of propagation provide extra degrees of freedom, significantly increasing the information capacity. The developed metasurfaces have the potential to impact many practical applications, including optical communications, dense data storage, and holographic encryption.

A metasurface can transform an incident beam with *l* = 0 into a series of discrete and equally spaced OAM modes, simultaneously carrying multiple various types of OAM components. Figure 1 illustrates the schematic of the proposed metasurface designed to generate multicolored OAM combs along the light propagation path. When a metasurface is illuminated by an incident Gaussian beam with multiple wavelengths, three different colored OAM combs are observed at the predesigned observation planes. Any desired mode spacing can be achieved in the resulting OAM comb. The OAM spectrum of the generated OAM comb is measured by the intensity back conversion technique,^8,38^ where an opposite TC is impinged to detect a specific OAM mode in the form of a Gaussian spot. This method simplifies the complex task of measuring the helical phase front of an OAM beam by converting it back into a form that is easier to measure using standard intensity-based detection methods (e.g., a camera).

**Figure 1:**
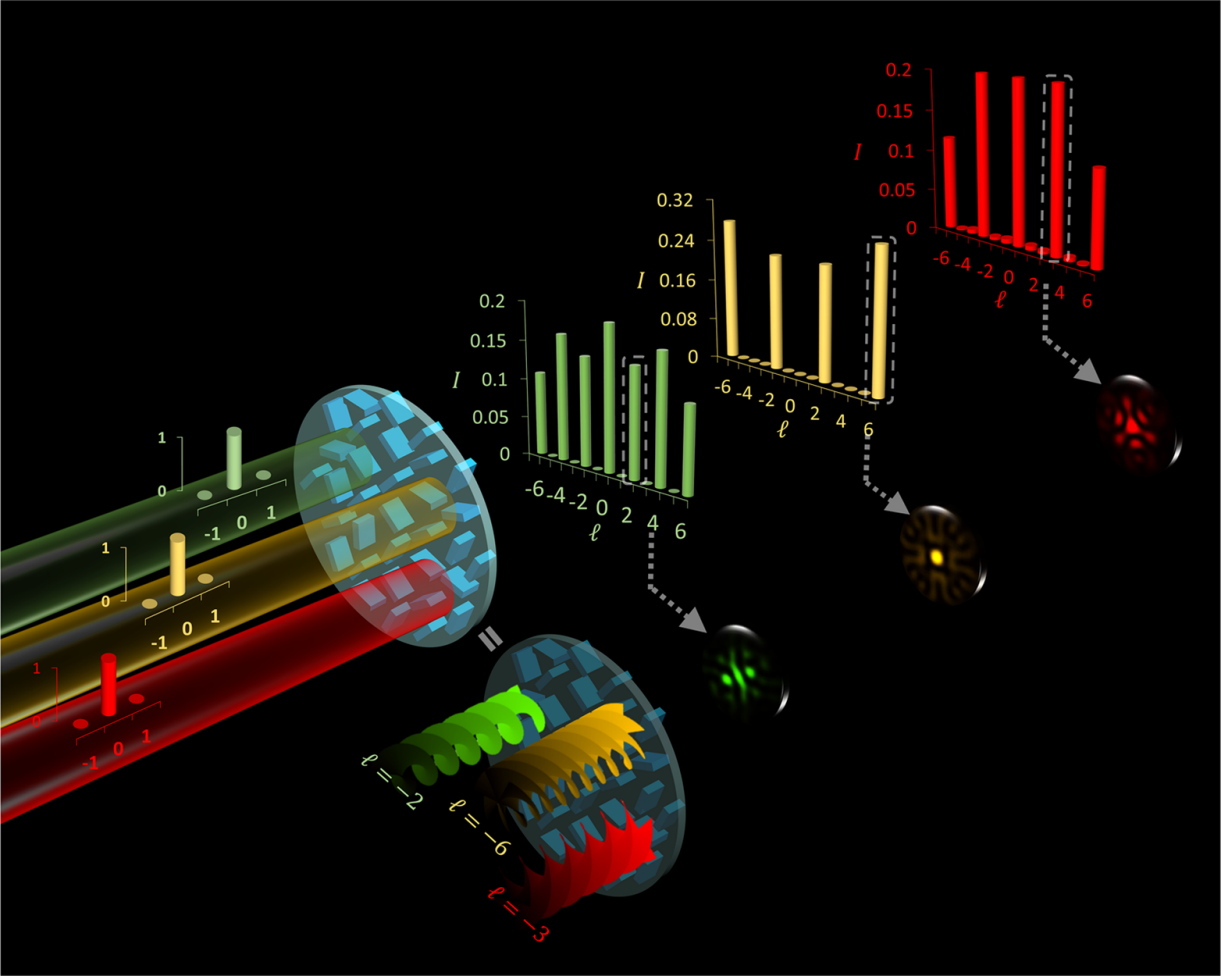
Schematic of a metasurface for realizing multicolored OAM combs. When illuminated by an incident Gaussian beam (*l* = 0) with three wavelengths (550, 600, 650 nm), a metasurface can generate OAM combs with different colors (green, yellow, red) at designated observation planes along the light propagation direction. Each OAM comb simultaneously carries a series of discrete and equally spaced OAM modes. The OAM spectra of the generated OAM combs are retrieved by the intensity back-conversion, where an incident OAM mode with an opposite TC is used to detect OAM modes, resulting in a Gaussian spot. For instance, to detect OAM modes +2 (green), +6 (yellow), and +3 (red) in different OAM combs, we must use OVs with TCs of *l* = −2, −6, and −3, respectively. The resultant intensity patterns are shown, featuring a bright Gaussian spot like intensity at the center.

Binary azimuthal phases are employed to realize such combs. A continuous azimuthal phase, also known as the spiral phase, θ_*spiral*_ = *lφ* can transform a Gaussian beam into an OAM beam with a TC *l*. Binarization of the spiral phase into 0 and π with a threshold of θ_*th*_ = π, as shown in Figure 2, results in simplified azimuthal binary phase grating, which can be written as
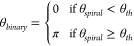
1θ_*binary*_ is the binary phase distribution. The OAM spectrum of the generated beam can be obtained through the OAM modal decomposition.^39,40^ The Kirchhoff diffraction theory^33^ is used to obtain the approximate far-field intensity by Fourier transformation of *E*_*binary*_ = *F*[e^(iθ_*binary*_)^]. *E*_*binary*_ can be decomposed into the sum of helical terms with various complex weights *A*_*l*_.^39^ Mathematically it can be expressed as
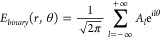
2
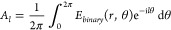
3Here, |*A*_*l*_|^2^ represents the intensity of corresponding OAM modes and *l* denotes a TC. As an example, we binarize the continuous azimuthal phases with one nodal line (*l* = 1) and two nodal lines (*l* = 2) as shown in Figure 2a,b, respectively. Their corresponding binary phases and OAM spectra are shown in the first column. Now, upon illumination of a Gaussian beam (*l* = 0), the resultant OAM spectrum contains multiple equally spaced OAM modes instead of a single OAM component. Moreover, the OAM spectrum can be further tuned by altering the θ_*th*_. To verify this, we incorporate an additional phase θ_*i*_ to manipulate θ_*th*_. The equation will become
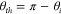
4

**Figure 2:**
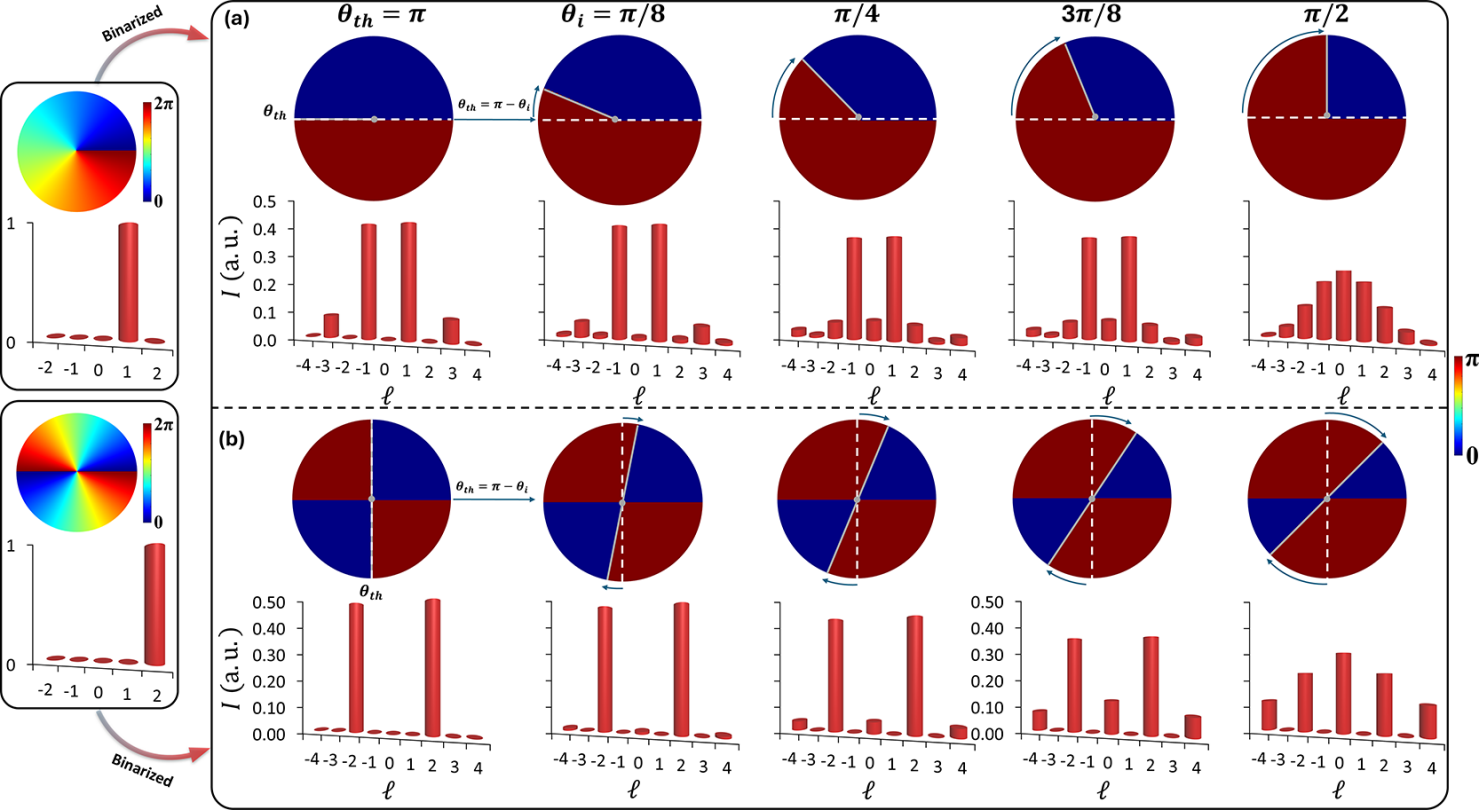
Working mechanism. Binarization of the spiral phase into 0 and π binary phase profiles. Binarized continuous azimuthal phases with (a) one nodal line (*l* = 1) and (b) two nodal lines (*l* = 2) with threshold θ_*th*_ = π (first column). The binarized phase produces new OAM spectra having fundamental OAM components at −1 and +1 in (a) and −2 and +2 in (b). The spectra can be further tuned by including different θ_*i*_ such as  (2nd column),  (3rd column),  (4th column), and  (5th column). In both (a) and (b), the top row indicates binarized phase profile, while the bottom row shows the corresponding OAM spectra.

To evaluate this effect, we choose different values of θ_*i*_, including , , , and . When θ_*i*_ is increased, a change in the spectrum can be observed. The corresponding intensity pattern for each spectrum is shown in Supplementary Section 1.

In general, there can be θ_*n*_ transition points in the individual binary phase, with the position of each determined by θ_*th*_. Different positions of the transition points yield a redistribution of the azimuthal phase, giving rise to a new OAM spectrum. The phase distribution of binary phase grating with *N* various azimuthal transition points can be mathematically written as
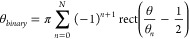
5where *n* = 0, 1, 2, ..., *N* is an integer and θ is an azimuthal angle. To experimentally realize this concept, we design and fabricate three different metasurfaces based on various transition points θ_*n*_. The acquired phase profiles are listed in Figure 3a. In the first design (left column of Figure 3a), we consider two transition points with θ_0_ = 0 and θ_1_ = 3.1416. In the second design (middle column of Figure 3a), four transition points are considered with θ_0_ = 0, θ_1_ = 1.5708, θ_2_ = 3.1416, and θ_3_ = 4.7124. In the third design (right column of Figure 3a), we chose 12 transition points with θ_0_ = 0, θ_1_ = 0.69, θ_2_ = 1.4, θ_3_ = 1.5708, θ_4_ = 2.2637, θ_5_ = 2.9708, θ_6_ = 3.1416, θ_7_ = 3.8345, θ_8_ = 4.5416, θ_9_ = 4.7124, θ_10_ = 5.4053, and θ_11_ = 6.1124. The design also incorporates a metalens model to get the focused intensity distribution. The corresponding optical microscope images of the fabricated metasurfaces with an area of 400 × 400 μm^2^ are shown in Figure 3b. The inset shows SEM images of metasurfaces consisting of silver (Ag) nanorods with different orientation angles sitting on an ITO-coated glass substrate. The length, width, and height of each nanorod are 200, 80, and 40 nm, respectively. The pixel size of the metasurface is 300 nm along both the *x*- and *y*-directions. The simulated conversion efficiency ranges from 8 to 11% (in the visible domain), which can be dramatically improved with dielectric metasurfaces. Detailed metasurface design information is provided in Supplementary Section 2. Details of the nanofabrication are provided in the Methods section.

**Figure 3:**
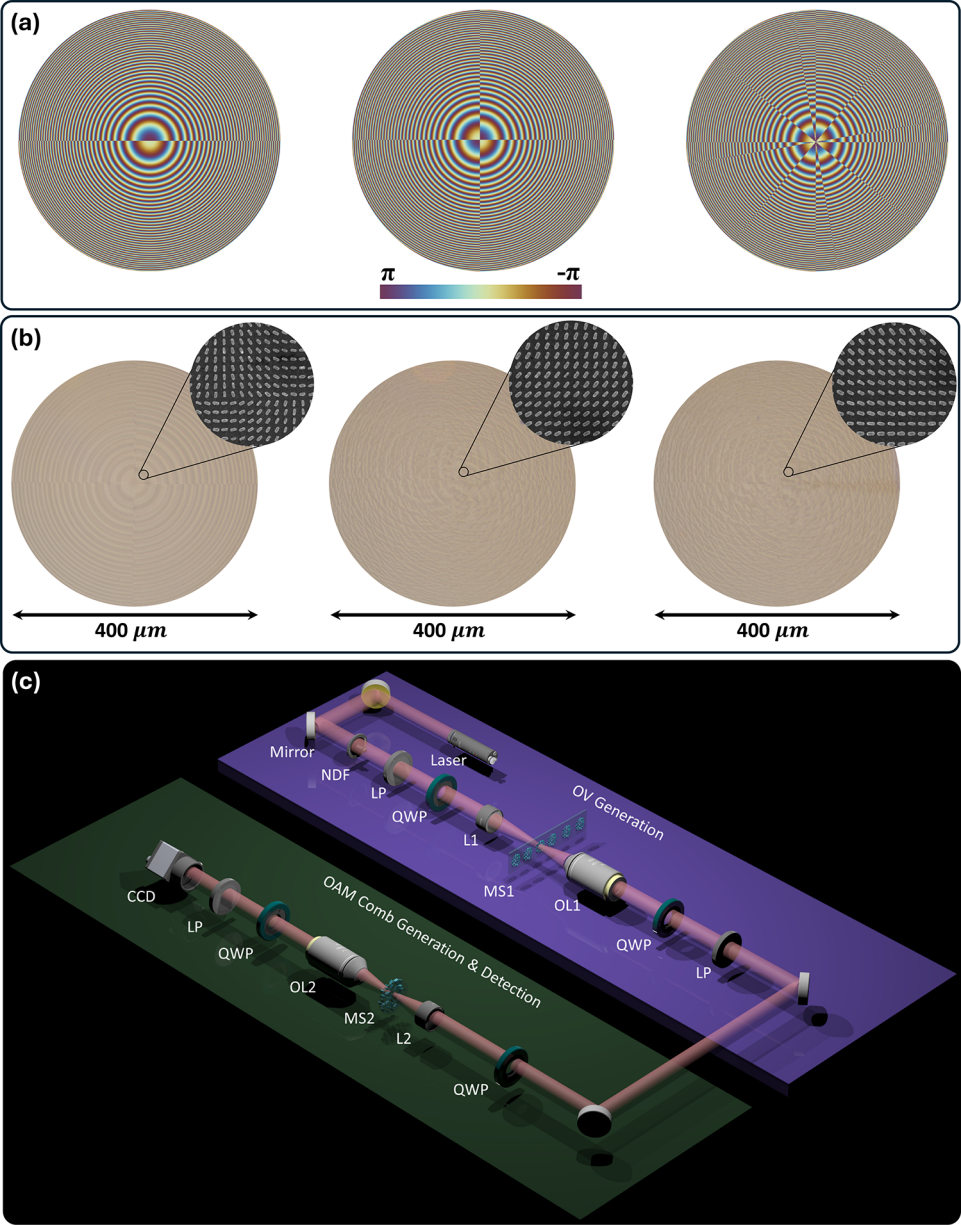
Nanofabrication and device characterization. (a) Designed phase profiles for three different OAM combs. (b) Optical microscope images of fabricated metasurfaces. The inset shows the SEM images of metasurfaces consisting of Ag nanorods with spatially variant orientations. (c) Schematic of the experimental setup. NDF: neutral density filter. LP: linear polarizer. QWP: quarter-wave plate. L1: lens with a focal length of 100 mm. L2: lens with a focal length of 75 mm. MS1: metasurfaces for generating OAM modes with TCs ranging from −6 to +6. MS2: metasurface for generating OAM combs, OL1: an objective lens with a magnification of 10×. OL2: an objective lens with a magnification of 50×. CCD: charge-coupled device.

The experimental configuration to characterize the fabricated samples is illustrated in Figure 3c. The NKT-SuperK EXTREME supercontinuum laser source is used to generate an incident light beam at 633 nm. The setup consists of two main parts: one for the generation of the OAM and the other for the generation and detection of the OAM comb generation and detection. In the first part, circular polarization is created with a linear polarizer (LP) and a quarter-wave plate (QWP). A lens (L1) with a focal length of 100 mm is employed to weakly focus the light onto the sample (MS1) (see Supplementary Section 3 for details). The MS1 contains six samples to generate the OAM beams with *l* = ±1, ±2, ±3, ±4, ±5, and ±6. A 10× objective lens (OL1) collects the generated OAM on the transmission side after the light beam passes through another QWP and LP. In the second part, the generated OAM is directed onto MS2 with the help of a QWP and a lens (L2) with a focal length of 75 mm. The OAM comb is then collected by a 50× objective lens (OL2). A CCD camera is used for visualization after removing the unconverted part through a QWP and an LP.

The intensity back conversion is used in the experiment to recover the OAM spectrum. In this technique, if an OAM comb has an *l* OAM mode, an incident light beam with −*l* yields a bright Gaussian spot at the center of an intensity pattern because opposite modes cancel each other (*l* – *l*_*in*_ = 0). Other OAM modes in the comb do not cancel out and thus do not produce a bright intensity spot at the center. Figure 4a shows simulated and experimentally obtained back-converted intensity patterns for the three different OAM combs designed in Figure 3. The small dashed circle indicates the central region where the bright Gaussian spot appears. In the image processing, this region is isolated using a grayscale algorithm. The collection area for this bright spot is defined by a circle with a diameter of 20 pixels, as shown in Figure S9. The key criterion for selecting this diameter is that it must completely encompass the central bright spot (fundamental mode only) without including any peripheral spots. The grayscale values of all pixels within the selected region of interest in the intensity profile captured by CCD are then summed. The main issue related to CCD measurement is distortion or sensitivity problems caused by overexposure, which can be avoided by maintaining the received intensity below the saturation threshold of CCD. While our method is effective and acceptable, alternative techniques such as OAM modal decomposition,^40^ the rotational Doppler effect,^41^ and log-polar coordinate transformation^42^ can also be used for OAM detection. It is worth noting that the size and position are fixed for the entire OAM spectrum. Due to the symmetrical nature of the OAM comb, the intensity patterns for only *l* = −6 to 0 are shown in Figure 4a. The intensities highlighted in red rectangular dashed boxes indicate the existence of the corresponding OAM mode as the bright spot appeared at the very center of the intensity profile. All other modes show a dark region inside the dashed circle. Figure 4b shows the bar graph to quantitatively evaluate the OAM content of combs. Figure 4b (left) shows four different OAM modes with TCs ranging from −3 to +3. Figure 4b (middle) displays two different OAM modes with TCs ranging from −2 to +2 and Figure 4b (right) presents four different OAM modes with TCs ranging from −6 to +6. It is important to note that the OAM spectra presented here show normalized intensities.

**Figure 4:**
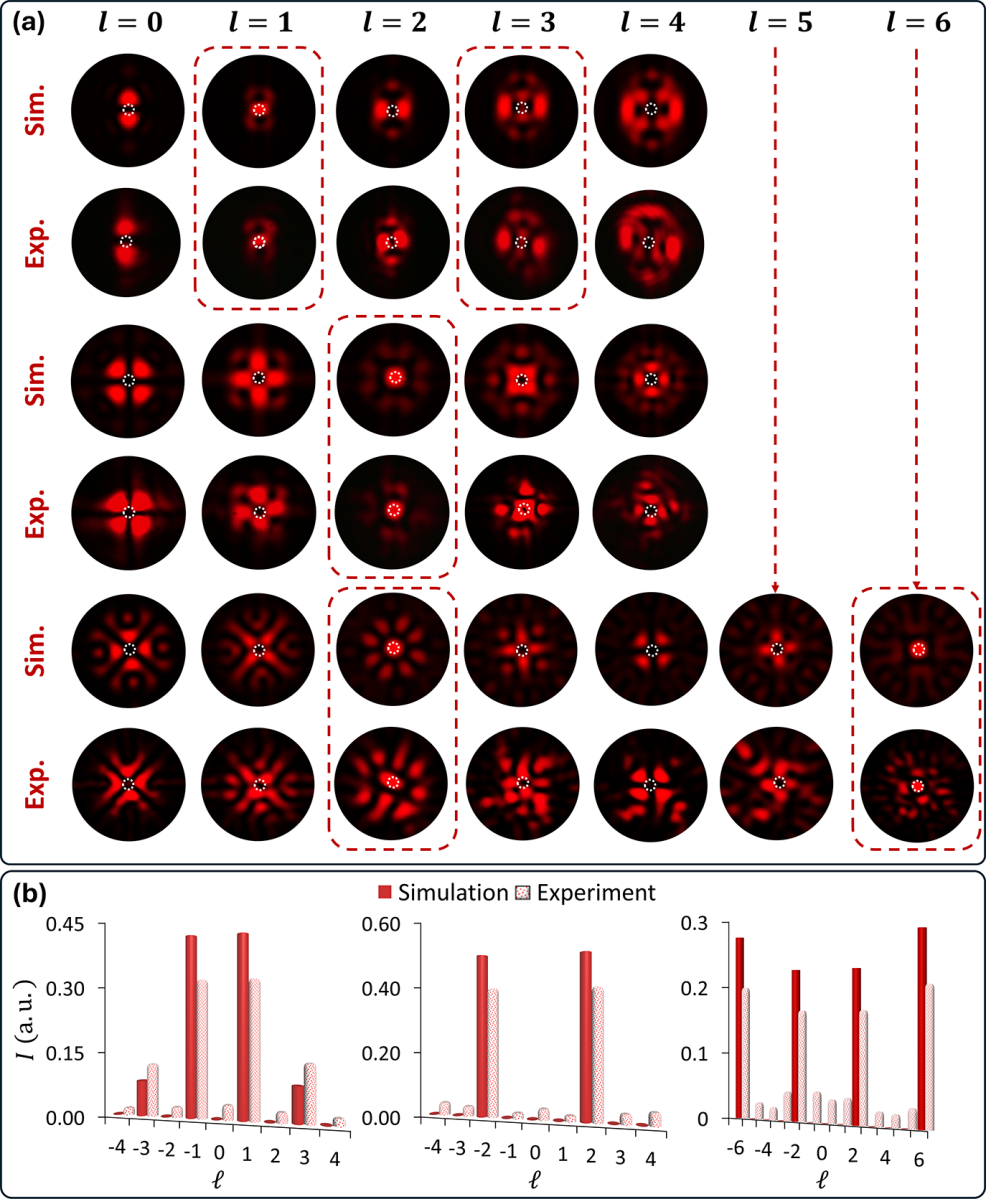
Experimental verification. (a) Simulated and measured intensity profiles for various OAM combs upon the illumination of incident light with different OAM modes. The presence of specific OAM modes is confirmed by the bright spot in the center, indicated by a dotted circle. Rectangular dashed lines highlight the existence of different OAM modes within the OAM combs. (b) Histograms show the simulated and experimental OAM spectra corresponding to the different intensities shown in (a). The varying weights of the bars reflect the differences in the brightness of the central spot. Left: Four OAM modes with TCs ranging from −3 to +3. Middle: Two OAM modes with TCs ranging from −2 to +2. Right: Four OAM modes with TCs ranging from −6 to +6.

To illustrate the versatility of our design, we present a more complex configuration of the OAM combs, featuring three different colors with various distributions of the OAM components. Figure 5a and Supplementary Section 4 show the detection of the OAM combs at three different predesigned observation planes (0.9, 1.3, and 1.7 mm) under circular polarization with three different colors (550, 600, and 650 nm). A multifoci metalens model is used to include spectral information in OAM combs. Mathematically, the metasurface design for *C* number of OAM combs can be expressed as
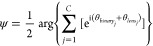
6where
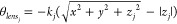
7Here *j* is an integer ranging from 1 to *C*.  is the *j*^*th*^ wavenumber, and λ*_j_* is the operating wavelength for *j*^*th*^ OAM combs at *z*_*j*_ focal point. The associated phase profile and an SEM image are provided in Supplementary Section 5. The dashed rectangular boxes indicate the existence of the corresponding OAM mode for a particular color. Figure 5b shows the complete OAM spectra of multicolored OAM combs at different *z*-planes. The green, yellow, and red colors contain 7, 4, and 5 completely different OAM modes with TCs ranging from −6 to +6, respectively.

**Figure 5:**
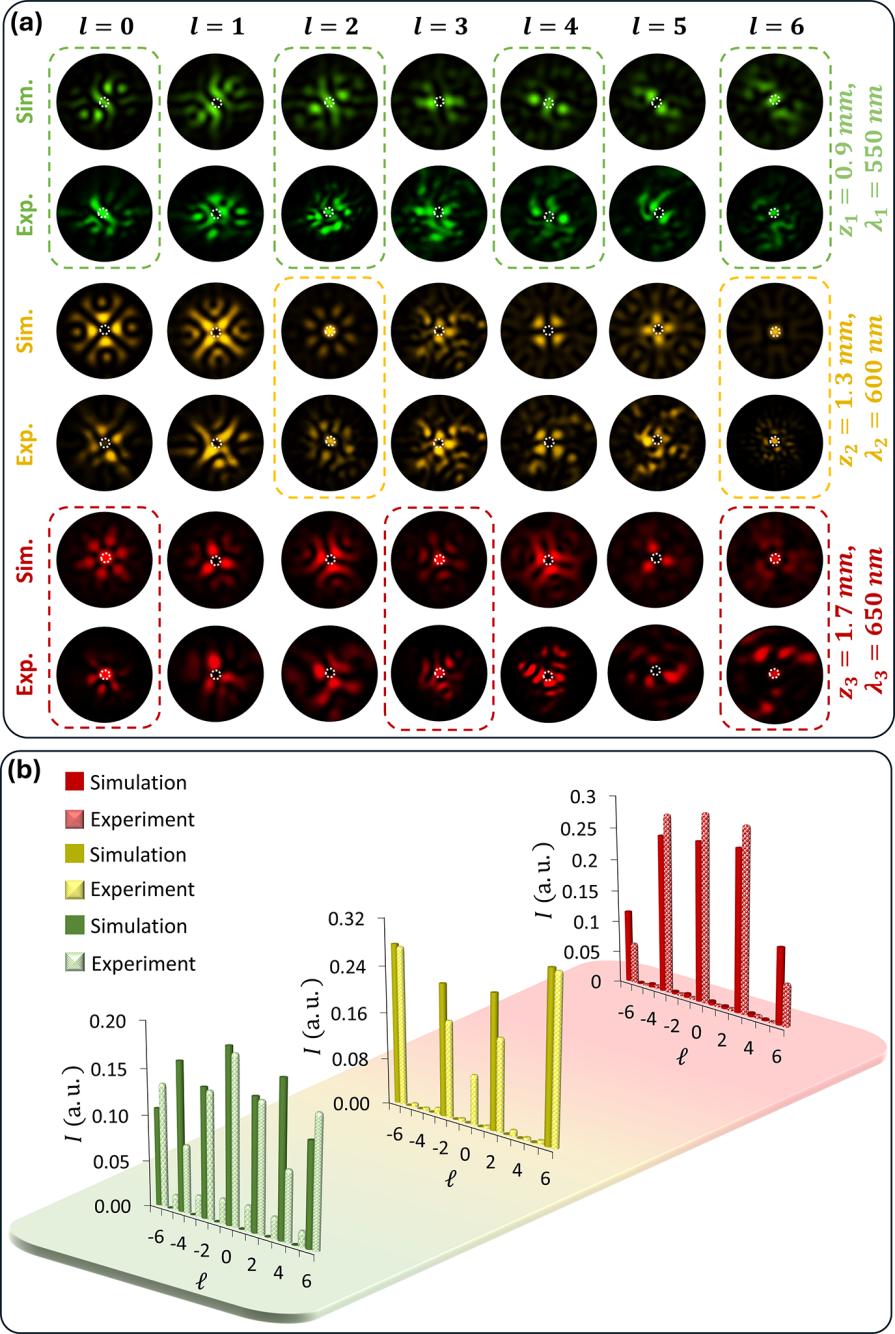
Quantitative analysis of multicolored OAM combs. (a) Simulated and measured intensity profiles of multicolored OAM combs under the illumination of incident light with various OAM modes. The metasurface generates three OAM combs (550, 600, and 650 nm) at distinct observation planes (0.9, 1.3, and 1.7 mm) along the light propagation on the transmission side. Specific OAM modes are identified by a bright spot in the center, indicated by a dotted circle. Colored dashed rectangles highlight different OAM modes at corresponding wavelengths within the OAM combs. (b) Histograms illustrate the complex OAM spectra (simulated and experimental) for three OAM combs with different wavelengths observed at three observation planes along light propagation. The varying heights of the bars indicate differences in the brightness of the central spots. The green (550 nm), yellow (600 nm), and red (650 nm) combs feature 6, 4, and 5 OAM modes, respectively. The OAM modes include TCs ranging from −6 to +6.

Like frequency combs, the OAM combs carry many OAM modes simultaneously. An azimuthal binary phase grating is used to transform an incident Gaussian beam (*l* = 0) into a structured beam that contains the desired OAM components. The position and number of thresholds/transition points (θ_*th*_, θ_*n*_) are carefully controlled to obtain and tune the desired OAM combs (as illustrated in Figure 2) with great flexibility. The intensity back-conversion technique is used to experimentally verify the combs’ generation in the form of bright spots (Supplementary Section 6). A separate metasurface is used to generate orthogonal OAM modes for detection. A more detailed explanation of the design methodology and purity of the generated OAM modes is provided in Supplementary Section 3. The brightness of the spot depends on the weight of the OAM mode involved, i.e., the higher the weight, the brighter the spot, and vice versa (as shown in Figure 4 and Figure 5a). We also demonstrate the manipulation of the OAM comb using the scaling property of the Fourier transform, with varying mode intervals and a multicolored design (Supplementary Section 7). By adjusting the scaling factor, we can control the OAM spectrum across different TC distributions. Additionally, multicolored OAM combs are presented with different shifting factors (Supplementary Section 8). Shifts in the OAM spectrum occur at predefined observation planes for specific wavelengths, illustrating the effects of right and left shifts by factors of +2 and −3, respectively. Although the number of wavelengths in the OAM comb demonstration is three in this work, it can be further improved by increasing the number of pixels and reducing the pixel size of metasurfaces. We also design a metasurface to generate three OAM combs with different wavelengths in the same observation plane (See Supplementary Section 9). As a proof-of-concept demonstration, we utilize a plasmonic (silver (Ag) nanorod-based) metasurface with a low conversion efficiency. In comparison with previously demonstrated OAM combs, a comparison table in terms of efficiency and multispectral information is provided in Supplementary Section 10. This efficiency issue can be solved with a dielectric metasurface.^26^ Since the generated beam is a type of structured light beam, the proposed concept can be extended to realize spatially variant vector OAM combs, Laguerre-Gaussian (LG) and Hermite-Gaussian (HG) combs, as well as nondiffracting Bessel combs. We propose a generalized metasurface approach to simultaneously generate and manipulate multiple OAM combs with different wavelengths. The unusual properties of metasurfaces in this research offer extraordinary capabilities in a variety of applications, including high-security holographic encryption and a high-dimensional data communication. Multicolored OAM combs at various predefined positions introduce unique properties, opening new opportunities for practical applications. For instance, in OAM multicasting, the spatial separation of different OAM combs enables efficient multiplexed data transmission, significantly enhancing the information capacity of OAM-based optical communications.^7^ The ability to control the position and wavelength of OAM modes further enables advanced holographic encryption and decryption, where an OAM comb serves as a flexible key for secure data storage and retrieval.^9^ Additionally, in quantum computing and cryptography, the use of OAM combs can create multidimensional photon entanglement, improving security and efficiency in quantum communications.

In conclusion, we have successfully demonstrated the generation and manipulation of OAM combs with a single metasurface. Multicolored OAM combs with intricate OAM spectra at predefined observation planes are observed based on azimuthal binary phase gratings. This novel approach overcomes the limitations of traditional OAM comb generation techniques, offering a noniterative, compact, and flexible solution. The ability to control the position and number of transition points within the spiral phase enables precise engineering of the OAM combs, significantly enhancing their information capacity and potential applications. The implications of this work are far-reaching with potential applications in optical communication, dense data storage, and high-security holographic encryption. Our metasurface-based method establishes a new standard for developing advanced OAM comb systems, unlocking new possibilities for high-dimensional data communication and a range of innovative applications.

## Methods

The designed metasurfaces are composed of Ag nanorods on a glass substrate. Electron beam lithography and the lift-off process are used to fabricate proposed metasurfaces. We use the positive tone electron beam resist poly(methyl methacrylate) (PMMA) 950 for the electron beam patterning. Initially, a 120 nm thick PMMA film is spin-coated onto an ITO-coated glass substrate and baked on a hot plate at 130 °C for 3 min. The PMMA resist is then exposed to an electron beam with an accelerating voltage of 30 kV and a beam current of 36 pA. The PMMA patterns are developed by immersing the sample in the mixture of methyl isobutyl ketone: isopropyl alcohol (1:3) for 75 s, followed by isopropyl alcohol for 60 s. Subsequently, a 40 nm thick Ag film is deposited on the sample using an electron beam evaporator. Finally, the Ag nanorods are obtained for characterization after the lift-off process in acetone.

## Supporting Information Available

Investigating the effect of θ_*i*_, metasurface design, metasurfaces for generating optical vortex beams, multicolored OAM combs, multicolored comb design, OAM comb detection, scaling OAM spectrum, shifting OAM spectrum, multicolor OAM combs at single observation plane, efficiency and multispectral capabilities of various methodologies, Kirchhoff–Fresnel diffraction integration for multicolored OAM combs (PDF)
