# Supplementary material for: Customizing Multicolored Orbital Angular Momentum Combs
Source: Nano Lett. 2025 Mar 24;25(13):5366–74. doi: 10.1021/acs.nanolett.5c00467 (PMC11969648; doi:10.1021/acs.nanolett.5c00467)
Supplement: Supplementary file 1 — nl5c00467_si_001.pdf [file nl5c00467_si_001.pdf]

Supporting Information

Customizing multicolored orbital angular momentum combs

Hammad Ahmed[1], Muhammad Afnan Ansari[1], Rong Yan[1,2], Xianzhong Chen[1,]*

[1]Institute of Photonics and Quantum Sciences, School of Engineering and Physical Sciences, Heriot-Watt University, Edinburgh EH14 4AS, UK
E-mail: x.chen@hw.ac.uk

[2]MIIT Key Laboratory of Complex-field Intelligent Sensing, Beijing Institute of Technology, Beijing 100081, China

Supplementary Section 1: Investigating the effect of θ_i

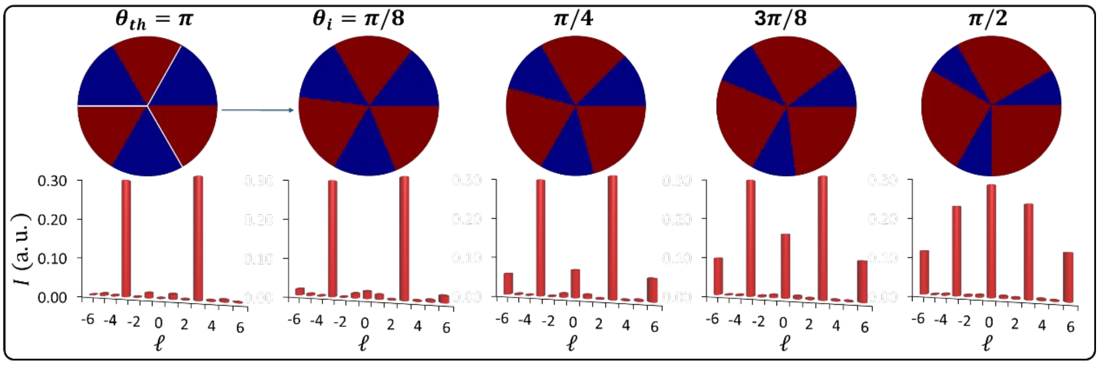

Figure S1. Binarisation of the spiral phase into 0 and π binary phase profiles. Binarized continuous azimuthal phases with three nodal line ($l = 3$) with threshold θ_{th} $= \pi$ (1st column). The binarized phase produces new OAM spectra having OAM components at -6 to +6. Further tuning of spectra is performed by incorporating various θ_i such as $\frac{\pi}{8}$ (2nd column), $\frac{\pi}{4}$(3rd column),$\frac{3\pi}{8}$ (4th column), and$\frac{\pi}{2}$ (5th column). The corresponding intensity pattern for each spectrum is shown in **Figure S2c**.

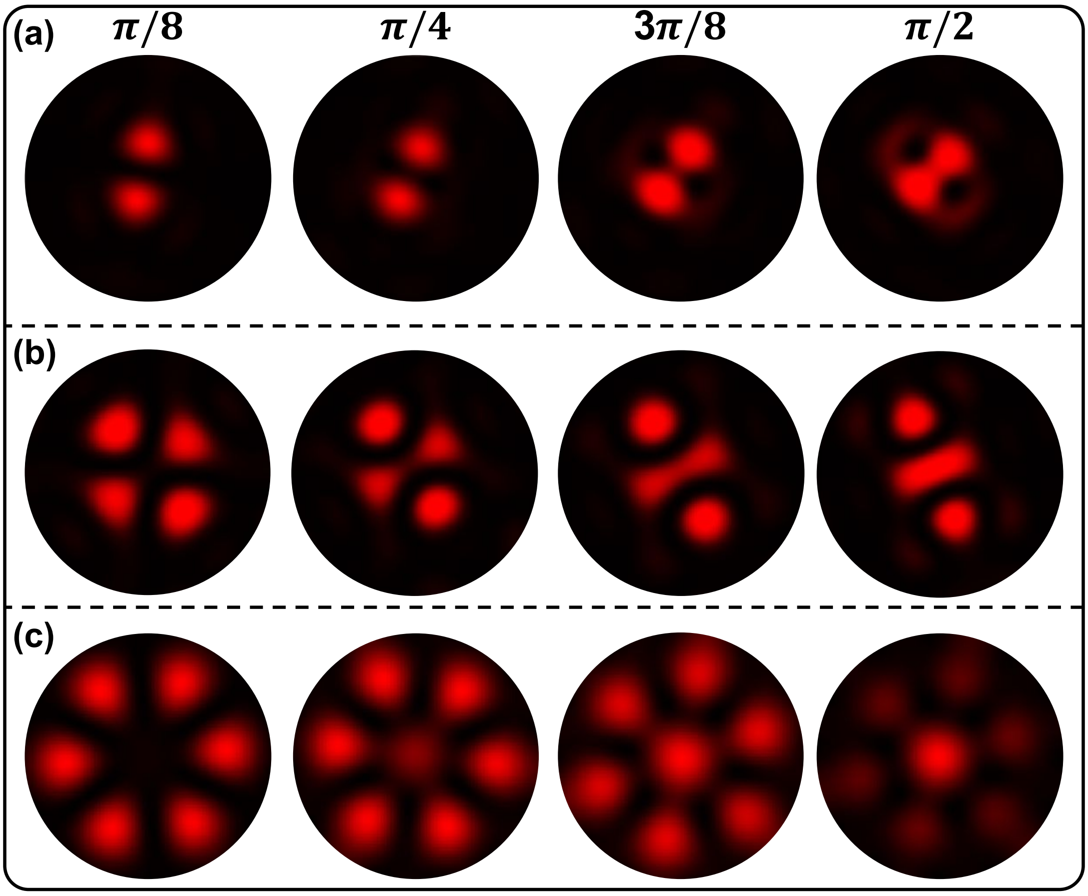

Figure S2. Farfield intensity distributions by adding various θ_i. Intensity distribution of binarized continuous azimuthal phases with **(a)** one nodal line (l=1), **(b)**

two nodal lines (l=2) and **(c)** threer nodal lines (l=3). Various intensity distributions yield distinct OAM spectra as shown in Figure 2 and S1.

Supplementary Section 2: Metasurface design

All the metasurface devices in this manuscript consist of silver (Ag) nanorods with different orientations on a glass (SiO_2) substrate, as shown in **Figure 3b**. The schematic of the meta-atom (nanorod) is presented in the inset of **Figure S3**. Each nanorod has dimensions of L = 200 nm (length), W = 80 nm (width), and H = 40 nm (height). The pixel size is P = 300 nm along both the x- and y-directions. **Figure S3** shows the simulated conversion efficiency, which is calculated using the frequency-domain solver of the Computer Simulation Technology (CST) Microwave Studio software. The permittivity of the silver is defined by the Drude model, and the refractive index of the glass substrate is 1.46. A unit cell boundary condition is applied along the x- and y-directions, while an open boundary condition is applied along the z-direction. The calculated conversion efficiency remains relatively flat and uniform within the design band, ranging from 500 nm to 700 nm. The unconverted part is filtered out with the experimental setup shown in **Figure 3c**. Furthermore, the conversion efficiency can be dramatically improved with dielectric metasurfaces[1].

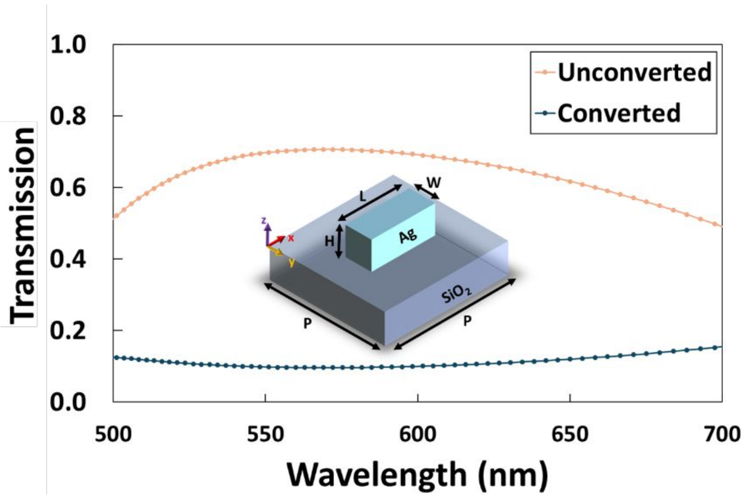

Figure S3. Meta-atom design. Simulated converted and unconverted transmission efficiency. The inset illustrates the schematic of Ag nanorod on a SiO_2 substrate. Length (L = 200 nm), width (W = 80 nm), height (H = 40 nm), and pixel size (P = 300 nm) are geometric parameters.

Supplementary Section 3: Metasurfaces for generating optical vortex beams

To generate various OAM modes, we fabricate six metasurfaces on a separate substrate to produce OAM modes with TCs ranging from -6 to 6, as shown in **Figure S4**. The experimental setup for OAM generation is shown in **Figure S5**. When a light beam impinges on a sample to generate the required OAM modes for OAM comb detection, the output contains both cross-polarized and co-polarized light. The non-converted portion can be removed using a second pair of a QWP and a LP[2]**.** Furthermore, interference patterns are produced by adjusting the angle of the output LP. **Figure S4a** shows the phase distributions for each of the six metasurfaces. **Figure S4b** presents SEM images of the fabricated samples, while **Figure S4c** displays the doughnut-shaped intensity patterns of the beams produced by these samples. As the topological charge increases, the radius of the intensity ring also increases. **Figures S4d** and **S4e** illustrate the interference patterns recorded for left-circularly polarized (LCP) and right-circularly polarized (RCP) light, respectively, confirming the various TCs.

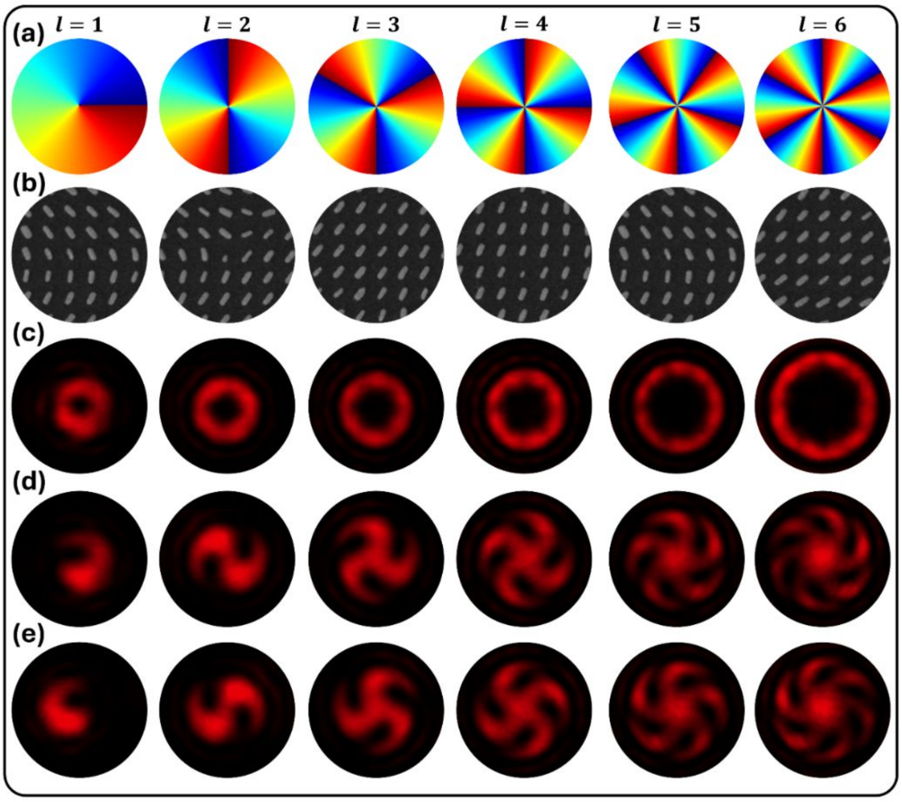

Figure S4. Metasurface design for generating OVs with $l = 1 - \pm 6$. (a) Phase distribution **(b)** SEM images of fabricated samples. **(c)** Measured doughnut shaped

intensity profiles for $l = 1 - 6$ under circular polarization (CP). **(d)** and **(e)** Interference pattern under Right circular polarization and left circular polarization, respectively. Number of petals determine the TC and direction indicate sign of TC.

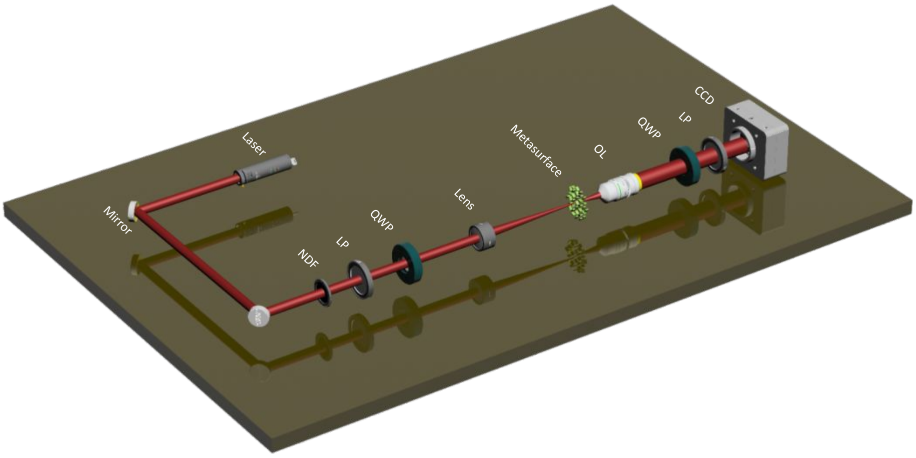

Figure S5. Experimental setup for generating OAM modes

Moreover we also calculate the OAM mode purity (**Figure S6**) using Modal decomposition method[3].

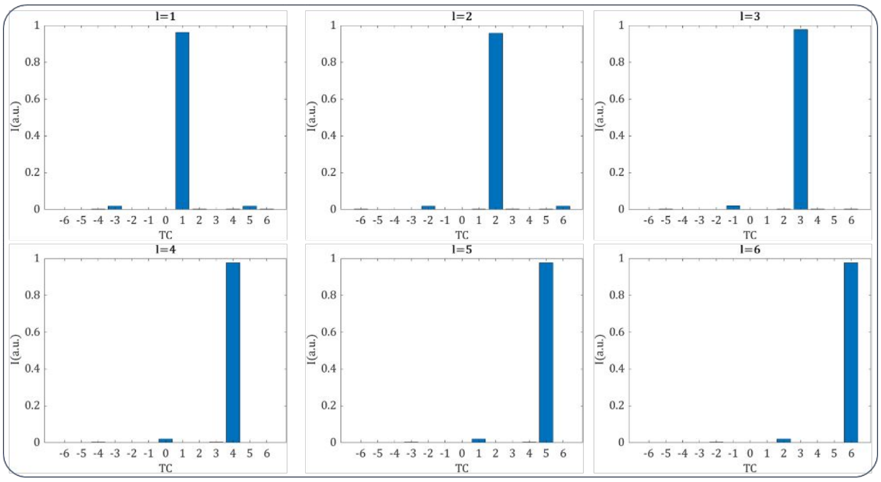

Figure S6. OAM mode Purity with different TCs.

Supplementary Section 4: Multicolored OAM combs

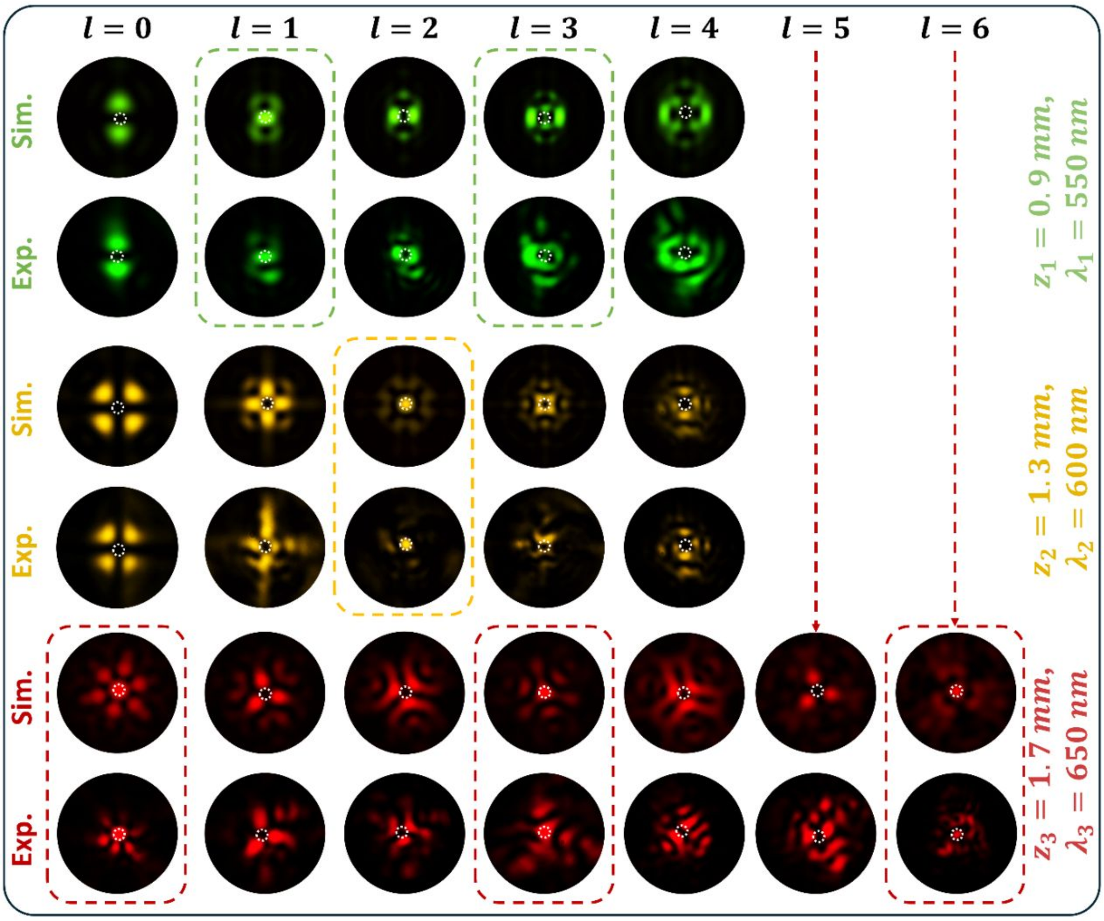

Figure S7. Multicolored OAM combs. Multicolored OAM combs along the light propagation. Simulated and measured intensity profiles of multicolored OAM combs under the illumination of incident light with various OAM modes. The metasurface generates three OAM combs (550, 600, and 650 nm) at distinct observation planes (0.9, 1.3, and 1.7 mm) along the light propagation on the transmission side. Specific OAM modes are identified by a bright spot in the centre, indicated by a dotted circle. Colored dashed rectangles highlight different OAM modes at corresponding wavelengths within the OAM combs. The associated phase profile and an SEM image are provided in **Supplementary Section 5**.

Supplementary Section 5: Multicolored comb design

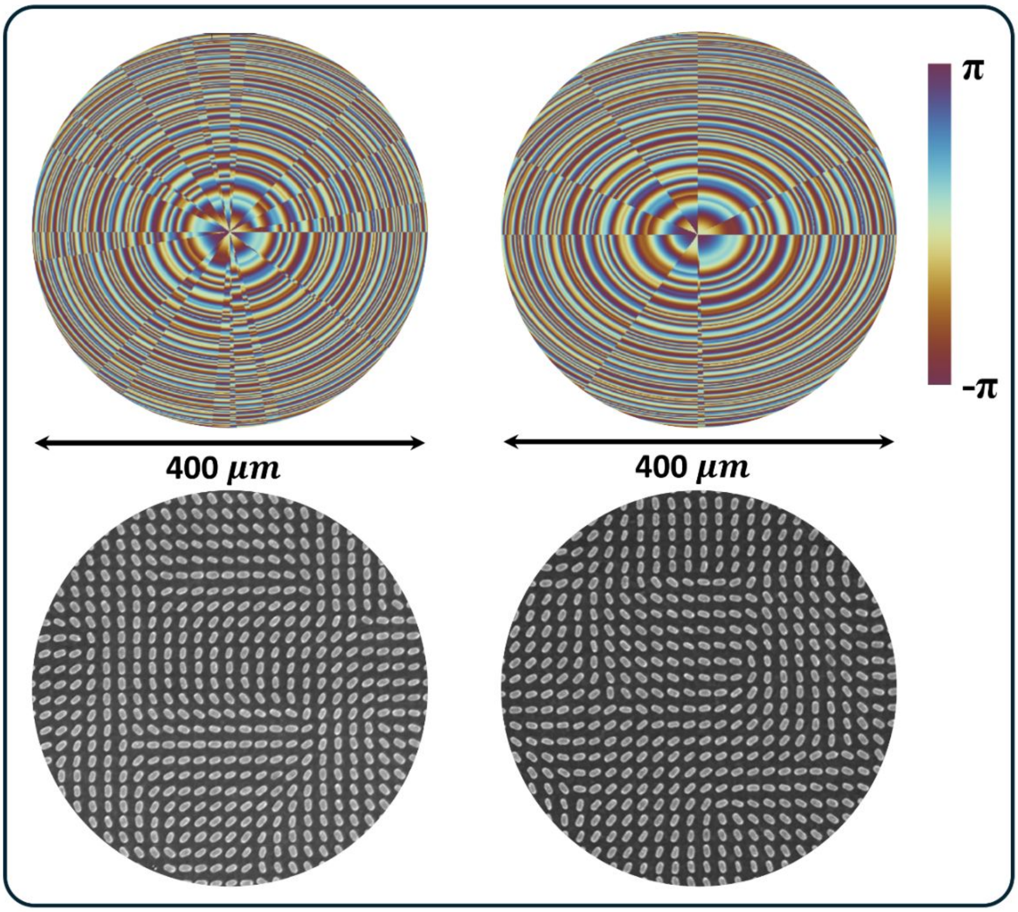

Figure S8. Metasurfaces for multicolored OAM combs. 1st row shows designed phase profiles for various multicolored OAM combs shown in **Figure 5** and **S7**. 2nd row shows the corresponding SEM images of the fabricated samples.

Supplementary Section 6: OAM comb detection.

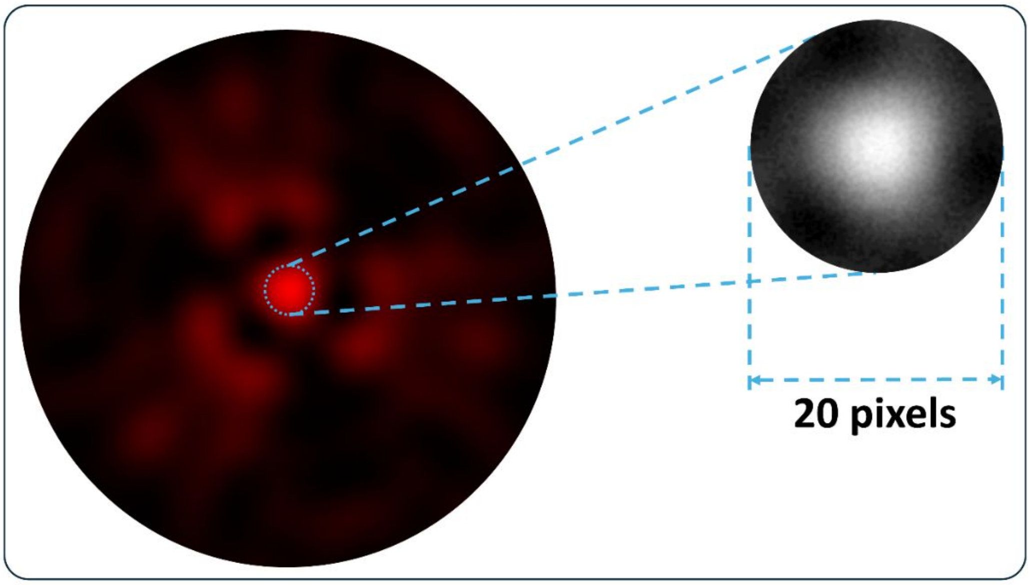

Figure S9. OAM mode detection. Intensity profile for OAM combs upon the illumination of incident light with OAM modes $l = -6$. The presence of specific OAM modes is confirmed by the bright spot in the centre, indicated by a dotted circle. The inset shows a zoomed-in version of the central region, illustrating the dimensions used for calculating the OAM spectrum.

In this work, the RGB intensity profiles captured by the CCD camera are used to illustrate various multicolored OAM combs. In the image processing **(Figure S9),** the central bright spot is isolated using a grayscale algorithm. The collection area for this bright spot is defined as a circle with a diameter of 20 pixels, as shown in **Figure S9**. The key criterion for selecting this diameter is that it must completely encompass the central bright spot (fundamental mode only) without including any peripheral spots. This selection is based on the principle of back conversion. When the incident beam is an optical vortex with a TC of $l = -1$, the OAM state $l = 1$ in an OAM comb will become 1 - 1 = 0, consequently, a bright spot appears at the center. We acknowledge that the bright region's apparent size and position may vary slightly in different images due to experimental and simulation conditions. However, we apply the same selection criterion consistently across all acquired intensity profiles to ensure a fair comparison.

Supplementary Section 7: Scaling OAM spectrum

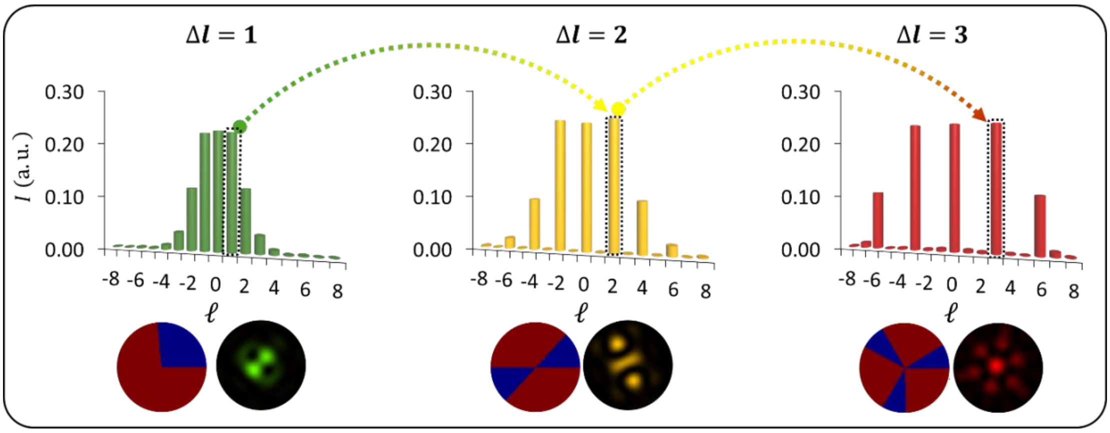

Figure S10. OAM comb with different mode interval. Multicolored OAM combs along the light propagation are shown with various mode intervals $\Delta l = 1 - 3$. The bottom row shows the corresponding phase and intensity profiles. The black dashed rectangles indicate an example of a single OAM mode scaling to TCs.

We performed OAM comb manipulation based on the scaling property of the Fourier transform ($F\{f(\varphi/\Delta l)\} = \Delta l F(\Delta l \varphi)$) with Δl being a scaling factor. As an example, we scaled azimuthal angle with 3 different scaling factor ranging from $\Delta l = 1$ to 3. A multicolored design, as shown in **Figure S10**, is used to implement scaling in light propagation with three different colors simulatanously. An OAM comb with seven different modes are chosen and their TC distribution vary based on the scaling factor. For instance, First column has a mode distrubtion from -3 to +3 with $\Delta l = 1$. The second column has a mode distrubtion -6 to +6 with $\Delta l = 2$. While the third column has mode a distrubtion -9 to +9 with $\Delta l = 3$. Highlighted modes can be seen as an example which scaled up from $l = +1$ to $+2$ and $+3$. This scalable manipulation of OAM modes enables efficient control of OAM spectrum which can be applied to areas such as optical communications, quantum information processing.

Supplementary Section 8: Shifting OAM spectrum

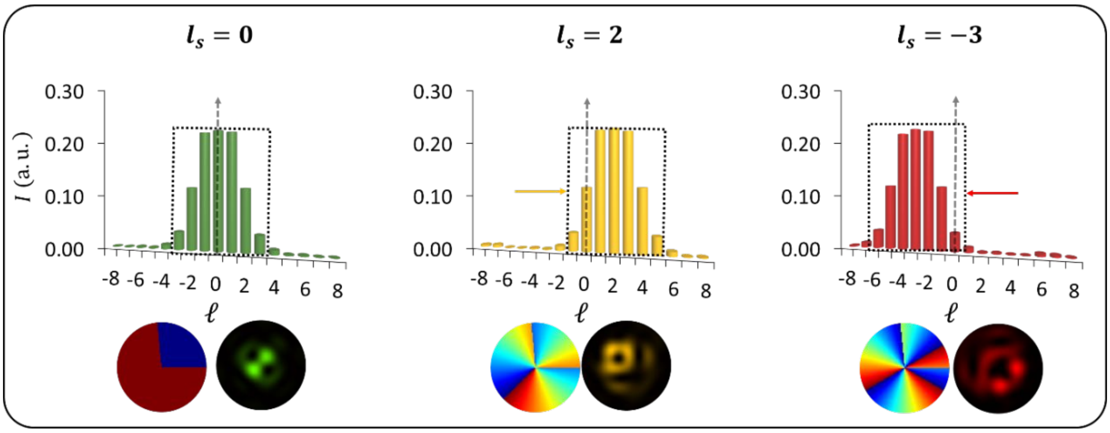

Figure S11. OAM comb with different mode interval. Multicolored OAM combs along the light propagation are shown with various shifting factors $l_s = 0, 2$ and -3. The bottom row shows the corresponding phase and intensity profiles. The dashed arrows indicate reference position. The middle column shows a shift in the OAM spectrum to the right by a factor of 2, while in the third column, the spectrum shifts to the left by a factor of 3. Each shift occurs at various predefined observation planes for specific wavelengths.

Supplementary Section 9: Multicolor OAM combs at single observation plane

To investigate the possibility of multicolor OAM combs in a single plane, where multiple wavelengths focus simultaneously, we simulate a metasurface device with a size of $400\ \mu m \times 400\ \mu m$. An off-axis multi-foci metalens model is used to design the metasurface[4]. Mathematically, the combined design for C OAM combs at a single observation plane can be expressed as:

$$\varphi = \frac{1}{2} arg \left\{ \sum_{j=1}^{C} \left[e^{i(\theta_{binary_j} + \theta_{lens_j})} \right] \right\} \quad (1)$$

where

$$\theta_{lens_j} = -k_j \left(\sqrt{(x - x_j)^2 + (y - y_j)^2 + z^2} - |z^2 + x_j^2 + y_j^2| \right) \quad (2)$$

Where x_j and y_j are the coordinates of jth focal point on the observation plane at working wavelength λ_j. **Figure S12** shows the multispectral intensity profile acquired at $z = 200\ \mu m$. The design includes three wavelengths (500 nm, 550 nm, and 650 nm), each supporting 5, 4, and 2 distinct OAM modes, respectively, with TCs ranging from

-4 to 4.

Although the presented simulation results are consistent with the expected design, slight background noise is observed due to the simultaneous involvement of multiple wavelengths. This issue would require further dispersion engineering[5] of the metasurface design, which needs future work to explore multiwavelength optimization strategies to achieve a more robust design while maintaining high efficiency.

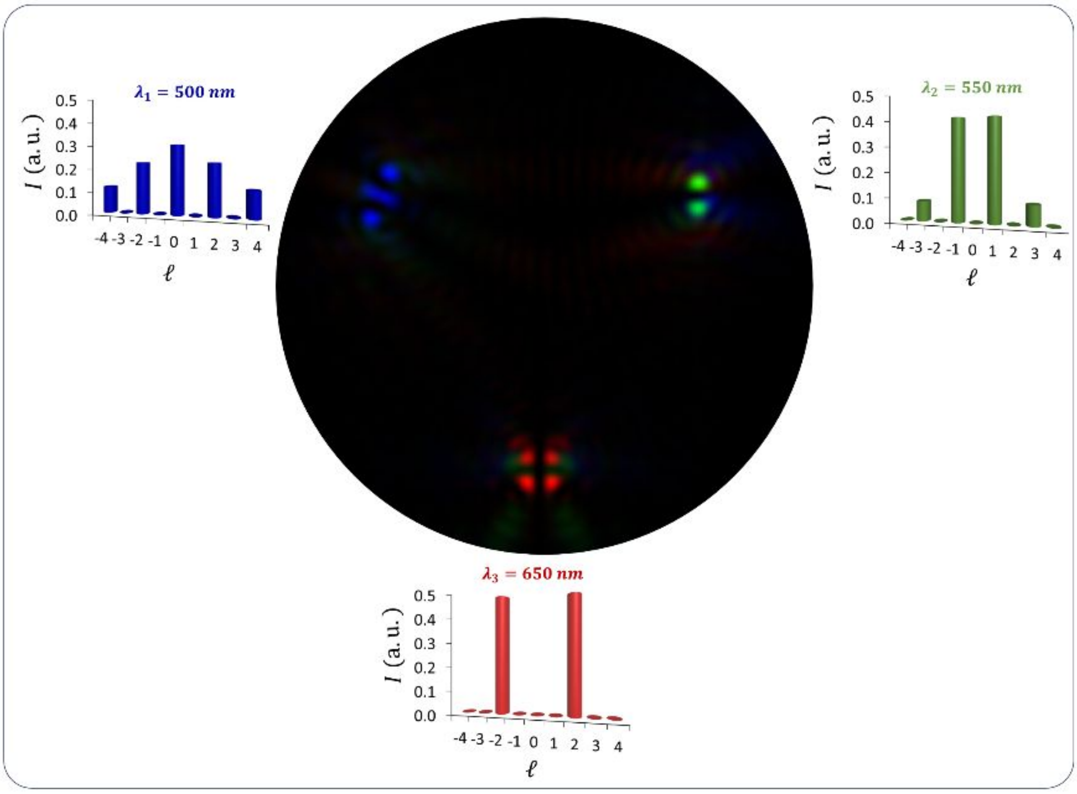

Figure S12. Multicolor OAM combs on a single observation plane at $z = 200\ \mu m$.

Supplementary Section 10: Efficiency and multispectral capabilities of various

methodologies

Table S1: Comparison of various methodologies.

Methodology	Efficiency	Multispectral Information
Adoptive Power Modification[6]	-	No
Pattern Search Algorithm (Simulation only)[7]	93%	No
Mode Iteration[8]	80%	No
Spatial Light Modulation[9]	60.19% - 83.31% (depending upon the number of modes involved)	No
Nested Ring Cavity[10]	0.55% - 7.1% (depending upon the number of modes involved)	No
Pinhole Plates[11]	-	No
THz Metasurface[12]	-	No
This work	8-11% (depending upon operating wavelength)	Yes

Supplementary Section 11: Kirchhoff–Fresnel diffraction integration for Multicolored OAM combs

All the proposed metadevices are simulated using the Kirchhoff–Fresnel diffraction integral. The actual size of the metasurfaces (400×400 μm^2) is used to calculate the intensity distributions at multiple observation planes, as described by the following equation [13, 14]:

$$E(x,y,z_n) = \sum_{n=1}^{N} \frac{e^{ik_n z_n}}{i\lambda_n z_n} \iint E(x_0,y_0,0) e^{-\frac{ik_n}{2z_n}[(x-x_o)^2+(y-y_o)^2]} dx_o dy_o \quad (S1)$$

Where, $n = 1, 2,\ldots, N$ is an integer representing the number of observation planes, E (x_0,y_0) is the initial amplitude profile of at the $z = 0$. x_o and y_o are the coordinates of nanorods, while x and y are the coordinates of the observation plane at a distance of z_n. $k_n = \frac{2\pi}{\lambda_n}$ is the wavevector for n^{th} observation plane and λ_n denotes the corresponding incident wavelength. the computation time depends on the resolution of the observation plane (i.e., the x, y grid). For this study, 100×100 μm^2 area on the

observation plane, with a resolution of 0.05 μm, is used.

References

1. Piccardo, M.; Ambrosio, A., Recent twists in twisted light: A Perspective on optical vortices from dielectric metasurfaces. *Applied Physics Letters* **2020,** *117* (14).

2. Ahmed, H.; Ansari, M. A.; Paterson, L.; Li, J.; Chen, X., Metasurface for Engineering Superimposed Ince-Gaussian Beams. *Adv. Mater.* **2024,** *36* (21), 2312853.

3. Pinnell, J.; Nape, I.; Sephton, B.; Cox, M. A.; Rodríguez-Fajardo, V.; Forbes, A., Modal analysis of structured light with spatial light modulators: a practical tutorial. *Journal of the Optical Society of America A* **2020,** *37* (11), C146-C160.

4. Ansari, M. A.; Ahmed, H.; Li, Y.; Wang, G.; Callaghan, J. E.; Wang, R.; Downing, J.; Chen, X., Multifaceted control of focal points along an arbitrary 3D curved trajectory. *Light: Science & Applications* **2024,** *13* (1), 224.

5. Chen, W. T.; Park, J.-S.; Marchioni, J.; Millay, S.; Yousef, K. M.; Capasso, F., Dispersion-engineered metasurfaces reaching broadband 90% relative diffraction efficiency. *Nat. Commun.* **2023,** *14* (1), 2544.

6. Li, S.; Wang, J., Adaptive power-controllable orbital angular momentum (OAM) multicasting. *Sci. Rep.* **2015,** *5* (1), 9677.

7. Zhu, L.; Wang, J., Simultaneous generation of multiple orbital angular momentum (OAM) modes using a single phase-only element. *Optics express* **2015,** *23* (20), 26221-26233.

8. Lin, J.; Yuan, X.-C.; Tao, S.; Burge, R., Collinear superposition of multiple helical beams generated by a single azimuthally modulated phase-only element. *Optics letters* **2005,** *30* (24), 3266-3268.

9. Fu, S.; Shang, Z.; Hai, L.; Huang, L.; Lv, Y.; Gao, C., Orbital angular momentum comb generation from azimuthal binary phases. *Advanced Photonics Nexus* **2022,** *1* (1), 016003-016003.

10. Ren, Z.-C.; Fan, L.; Cheng, Z.-M.; Liu, Z.-F.; Lou, Y.-C.; Huang, S.-Y.; Chen, C.; Li, Y.; Tu, C.; Ding, J., On-demand orbital angular momentum comb from a digital laser. *Optica* **2024,** *11* (7), 951-961.

11. Yang, Y.; Zhao, Q.; Liu, L.; Liu, Y.; Rosales-Guzmán, C.; Qiu, C.-w., Manipulation of orbital-angular-momentum spectrum using pinhole plates. *Physical Review Applied* **2019,** *12* (6), 064007.

12. Chong, M.-Z.; Zhou, Y.; Zhang, Z.-K.; Zhao, J.; Zhang, Y.-Y.; Zhang, C.-Q.;

Zang, X.; Du, C.-H.; Liu, P.-K.; Xia, M.-Y., Generation of polarization-multiplexed terahertz orbital angular momentum combs via all-silicon metasurfaces. *Light: Advanced Manufacturing* **2024,** *5* (3), 400-409.

13. Intaravanne, Y.; Wang, R.; Ahmed, H.; Ming, Y.; Zheng, Y.; Zhou, Z.-K.; Li, Z.; Chen, S.; Zhang, S.; Chen, X., Color-selective three-dimensional polarization structures. *Light: Science & Applications* **2022,** *11* (1), 302.

14. Zhang, Y.; Liu, W.; Gao, J.; Yang, X., Generating focused 3D perfect vortex beams by plasmonic metasurfaces. *Advanced Optical Materials* **2018,** *6* (4), 1701228.
